# Anticholinergics aggravate the imbalance of the autonomic nervous system in stable chronic obstructive pulmonary disease

**DOI:** 10.1186/s12890-019-0848-0

**Published:** 2019-05-09

**Authors:** Wei Yuan, Shan Nie, Haoyan Wang, Qiufen Xu, Nan Jia

**Affiliations:** Capital Medical University Affiliated Beijing Friendship Hospital, 95 Yongan Road Xicheng District, Beijing, 100050 China

**Keywords:** Anticholinergics, Heart rate recovery, Autonomic nervous imbalance, Chronic obstructive pulmonary disease

## Abstract

**Background:**

Inhaled anticholinergics, recommended as first-line maintenance treatment for patients with moderate-to-severe chronic obstructive pulmonary disease (COPD), has been demonstrated to be associated with an increased risk of cardiovascular diseases. Nevertheless, why COPD patients using inhaled anticholinergics have this higher risk remains unknown. One of mechanisms may be an autonomic imbalance because anticholinergics yield reduced vagal nervous activity. To test our hypothesis, we studied heart rate recovery (HRR) after exercise, recognized as a marker of cardiac autonomic function, in COPD patients using and not using inhaled anticholinergics.

**Methods:**

Sixty patients with COPD were involved in this study (mean FEV_1_ = 1.57 ± 0.42 L), including 24 patients who had received tiotropium for more than 1 year and 36 patients not using tiotropium as a control group. A maximal cardiopulmonary exercise test was performed. HRR was defined as the difference between peak exercise and at 1-min recovery heart rate.

**Results:**

HRR was significantly lower in patients using tiotropium than in the controls (16 ± 6 vs 22 ± 8 beats/min, respectively, *p* < 0.05). Multivariate regression analysis revealed that tiotropium use and peak VCO_2_ were independent predictors of HRR in these COPD patients.

**Conclusions:**

These findings suggest that anticholinergics bronchodilators reduce HRR after exercise in COPD patients. This has the potential to aggravate autonomic nervous imbalance. Therefore, we recommend that COPD patients taking anticholinergic bronchodilators should be considered for monitoring of cardiac function and prescribers should be alert for cardiovascular events that may arise from autonomic nervous imbalance.

## Background

Chronic obstructive pulmonary disease (COPD) is a largely preventable and manageable respiratory condition [1]. Bronchodilators are the mainstay of COPD management. Long-acting antimuscarinic antagonists (LAMAs, of which tiotropium bromide monohydrate, was the first available) have shown benefit for improving quality of life in COPD patients in large and randomized clinical trials (RCTs) [2, 3], They are currently recommended as first-line maintenance treatments for patients with moderate-to-severe COPD [2]. However, a population-based, nested case-control study of almost two hundred thousand patients with COPD found that new use of long-acting inhaled β- agonists and anticholinergics was associated with an increased risk of cardiovascular events [4]. Furthermore, the higher cardiovascular risk with inhaled anticholinergics has also been demonstrated in several randomized controlled trials and the meta-analyses of these trials [5, 6]. Nevertheless, the mechanisms linking anticholinergics to cardiovascular risk remains unknown.

One of mechanisms may be the autonomic imbalance. Sympathovagal balance is important for regulation of homeostasis, because the two nervous systems, sympathetic and parasympathetic (vagal), usually exert antagonist effects [7]. Studies showed that sympathetic overactivity and reduced parasympathetic activity were associated with ventricular arrhythmias and poor prognosis in patients with chronic heart failure [8]. Parasympathetic nerve fibres serve as components of the efferent limb of baroreceptors, chemoreceptors, and of other cardiovascular and respiratory reflexes involved in the regulation of cardiac automaticity and contractility [9]. Parasympathetic tone and parasympathetically mediated reflexes are profoundly depressed in heart failure and in various forms of heart disease. LAMAs affects cardiac function in two opposite ways [5]: First, anticholinergics relax bronchiolar smooth muscle, increasing ventilation and oxygenation. They indirectly decrease pulmonary artery pressure and compensatory sympathetic stimulation of the heart, reducing the incidence of tachyarrhythmia and ischemia. Second, anticholinergics suppress parasympathetic control of heart rate, associated with an increased incidence of tachyarrhythmia and myocardial ischemia [10]. Once the imbalance of the indirect and direct cardiac activity occurs, it may aggravate the autonomic nervous disorder.

Heart rate recovery (HRR) after exercise reflects parasympathetic reactivity and has been used as a marker of cardiac autonomic function [11]. Decreased HRR reflects lower parasympathetic activity. Decreased parasympathetic input to the heart is known to increase the potential for tachyarrhythmia and ischemia [5]. Several studies [11–13] have demonstrated that HRR was delayed in patients with abnormal spirometry, most of whom had COPD; abnormal HRR (≤12 beats) was a powerful predictor of overall mortality. However, to our knowledge, only a few studies have investigated whether an abnormal HRR was related to using of anticholinergics in patients with COPD; there is a particular lack of prospective research evidence. We hypothesized that anticholinergics would reduce vagal nervous activity. To test this hypothesis, we determined whether inhaled anticholinergic bronchodilators altered HRR in COPD patients.

## Methods

### Subjects

This was a prospective cohort study. COPD patients were recruited based on these inclusion criteria: 1) subjects aged 40–80 years; 2) the ratio of forced expiratory volume in the first second (FEV_1_) to forced vital capacity (FVC) < 0.7 and 30–80% of predicted FEV_1_ following 400 μg albuterol inhalation [1]; 3) being clinically stable at the enrollment of study, without respiratory infections or acute COPD exacerbations within last 6 weeks. The exclusion criteria were: 1) diagnoses of heart diseases, such as coronary artery disease, valvular disease, arrhythmia, heart failure; 2) receiving beta-blockers, non-dihydropyridine calcium channel blockers, or other heart rate modulating medications; 3) with temporary/permanent pacemakers or implantable defibrillators.

This study consisted of tiotropium group and controls. Patients in the tiotropium group had been on medical treatment with tiotropium bromide more than 1 year. The control group was COPD patients including patients who were either newly diagnosed or those who had not used tiotropium bromide, long-acting beta-agonists (LABAs), inhaled corticosteroids (ICS), or LABA/ICS in a single inhaler for the past 6 months. Subjects received written and verbal information explaining the study, and all patients gave written consent before participation. The study was approved by the Beijing Friendship Hospital ethics committee.

### Pulmonary function tests

ATS/ERS standards were followed for measuring FVC and FEV_1_ via spirometry [14]. All subjects were assessed through the use of a MasterScreen system (MasterScreen Body, CareFusion, Hoechberg, Germany) by a qualified test technician before exercise testing.

### Cardiopulmonary exercise test (CPET)

Lung function data were obtained within 2 weeks of exercise testing. Patients next underwent progressive incremental exercise testing to their symptom-limited maximum. All participants were not allowed to drink caffeine and smoke cigarette 2 h before the CPET. All of CPET were performed on a cycle ergometer with electronic braking (ViaSprint, CareFusion, Hoechberg, Germany). Following resting for 3 min, patients underwent 3 min of unloaded pedalling, and the incremental phase of exercise every minute at a 5 to 20 W/min work rate (individualized ramp protocol). All participants were encouraged to cycle for as long as possible until symptoms including leg fatigue, intolerable dyspnea, chest pain suggestive of ischemia, heart block, loss of coordination, dizziness, faintness and et al. prevented further exercise. Other termination criteria were:1) oxygen saturation via pulse oximetry (SpO_2_) ≤ 88%; 2) ventricular tachycardia; 3) ≥2 mm of horizontal or downsloping ST-segment depression; 4) blood pressure (BP) ≥ 240/130 mmHg; 5) a drop in systolic blood pressure (SBP) ≥ 20 mmHg from the highest value during exercise. A qualified exercise technician conducted each test with physician supervision. As heart rate recovery can be influenced by posture [15], subjects were instructed to sit on the ergometer and remain inactive throughout the recovery phase.

Subjects breathed through a mouthpiece. The parameters, such as oxygen uptake (VO_2_), carbon dioxide production (VCO_2_) and ventilation (V_E_), were acquired from breath-by-breath throughout the exercise test. Heart rate (HR), recorded from the ECG, and arterial oxygen saturation, estimated from pulse oximetry (SpO_2_), were also calculated breath-by-breath. Blood pressure was measured using a sphygmomanometer every 2 min. Variables were subsequently expressed as a 30-s average value for further analysis. The anaerobic threshold (AT) was established via a V-slope method. To estimate the cardiac function and exercise capability during exercise, we used the slope of HR/VO_2_、VO_2_/Watt and V_E_/VCO_2_ [16]. The V_E_/VCO_2_ slope was calculated by linear regression of V_E_ versus VCO_2_ from the beginning of exercise to the anaerobic threshold. The difference in HR at peak exercise and after a 1-min recovery period was used to define heart rate recovery (HRR).

### Statistical analysis

SPSS v19.0 was used for all analyses. Continuous variables were means ± SD, and were compared via paired t testing between subjects using and not using tiotropium bromide. Differences in abnormal HRR recovery proportions (≤12 beats) were compared using the chi-square test for trends. Statistical differences were significant at *P* value < 0.05. HRR determinants were assessed via a multivariate linear regression analysis.

## Results

Sixty patients with COPD were involved in this study; this included 24 patients using tiotropium and 36 patients control subjects. All patients enrolled were GOLD spirometry stage II-III, except for one stage IV in the control group. Table 1 presents the main anthropometric and lung function characteristics of the groups. No significant differences in age, gender, body mass index (BMI) or spirometric severity of COPD were detected between the groups.

**Table 1:** Anthropometric, functional characteristics between COPD with tiotropium and controls

Characteristic	Patients with tiotropium (*n* = 24)	Controls (*n* = 36)	*p* Value
Male, n (%)	20 (83.3)	34 (94.4)	–
Age (yr)	64 ± 9	65 ± 8	0.185
BMI (kg/m^2^)	24.48 ± 3.09	25.07 ± 3. 78	0.156
FVC (L)	2.73 ± 0.58	2.82 ± 0.60	0.648
FEV_1_ (L)	1.41 ± 0.36	1.67 ± 0.43	0.299
FEV_1_(% predicted)	50.26 ± 10.27	57.88 ± 13.45	0.069
FEV_1_/FVC (%)	51.81 ± 8.19	58.95 ± 6.77	0.238

Data are presented as mean ± SD

*COPD* chronic obstructive pulmonary disease, *BMI* body mass index, *FVC* forced vital capacity, *FEV*_*1*_ forced expiratory volume in the first second

**Fig. 1:**
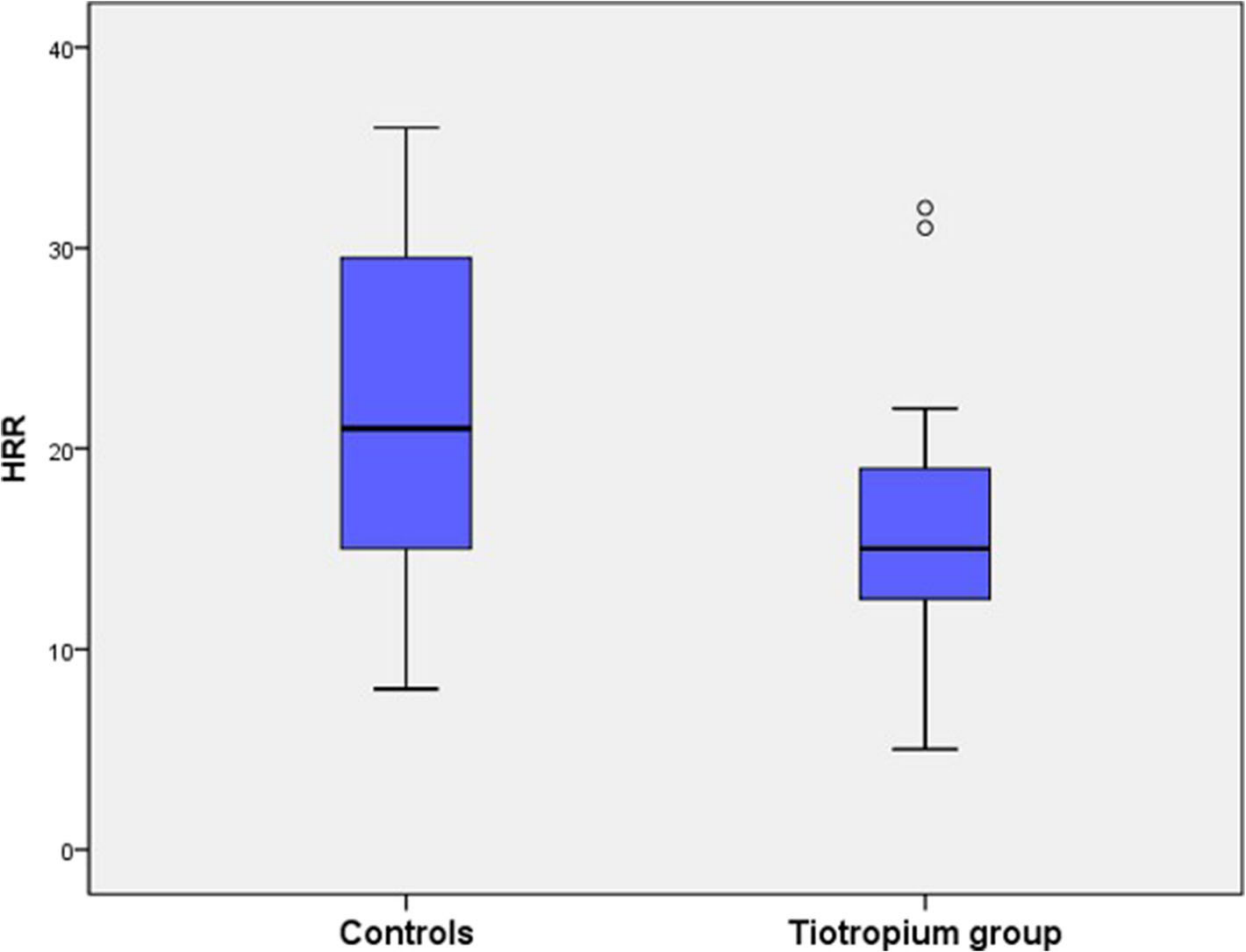
Heart rate recovery of using tiotropium group and controls. Paired t testing was used to compare HRR between subjects using tiotropium and controls. Mean of HRR was significantly lower in subjects using tiotropium than those in the control group (16 ± 6 vs 22 ± 8 beats/min, respectively, *p* < 0.05)

CPET data are presented in Table 2. Peak VCO_2_ and peak work rate were significantly higher in patients using tiotropium. Mean HRR was significantly lower in subjects with using tiotropium than in those in the control group (16 ± 6 vs. 22 ± 8 beats/min, respectively, *p* < 0.05) (Fig. 1). Peak VCO_2_ and peak-WR were significantly higher in the control group.

**Table 2:** CPET variables between COPD with tiotropium and controls

Variables	Patients with tiotropium (*n* = 24)	Controls (*n* = 36)	*p* Value
Peak WR (watt)	83.25 ± 23.19	102.64 ± 31.44	0.011*
Peak VO_2_(ml/min)	1155.25 ± 279.91	1409.19 ± 312.94	0.325
Peak VCO_2_(ml/min)	1326.63 ± 387.79	1594.31 ± 446.17	0.043*
AT (ml/min)	716.91 ± 286.70	946.44 ± 339.77	0.446
Mean of HRR (beats/min)	16 ± 6	22 ± 8	0.029*
VO_2_/WR slope (ml/min/watt)	8.37 ± 1.26	8.92 ± 1.29	0.800
HR/VO_2_ slope (beats/ml/min)	47.25 ± 12.71	47.54 ± 12.40	0.622
V_E_/VCO_2_ slope	29.57 ± 6.71	28.84 ± 7.33	0.923

*CPET* Cardiopulmonary Exercise Test, *COPD* chronic obstructive pulmonary disease, *WR* work rate, *VO*_*2*_ oxygen uptake, *VCO*_*2*_ carbon dioxide production, *V*_*E*_ minute ventilation, *AT* anaerobic threshold, *HRR* heart rate recovery

*significant difference, *P* < 0.05

Patients were dichotomized according to their HRR: normal (HRR>12 beats) and abnormal (HRR ≤ 12 beats). A total of 16.67% of subjects in the tiotropium group had abnormal HRR, compared to 13.89% in the control group. Chi-square analysis showed that there were no significant differences in abnormal HRR between the groups (*p* = 0.768).

Univariable correlation results are shown in Table 3. In the group as a whole, there was asignificant correlation with all pulmonary functions and exercise variables, except for V_E_/VCO_2_ slope. Multivariable regression analysis revealed that membership in the tiotropium group and peak VCO_2_ were the only variables that significantly entered the regression as predicting HRR (Table 4). The prediction equation from the multivariable regression analysis equation was *Y* = 12.641–4.327X_1_ + 0.006X_2_ (*Y* = HRR, *X*_*1*_ = membership in the tiotropium group, and *X*_*2*_ = peak VCO_2_).

**Table 3:** Correlation of HRR and key variables of pulmonary function and CPET

Variables	*r* Value	*p* Value
FEV_1_ (L)	0.352	0.006*
FEV_1_(%predicted)	0.322	0.012*
FEV_1_/FVC (%)	0.395	0.002*
Peak WR (watt)	0.477	0.000*
Peak VO_2_(ml/min)	0.482	0.000*
Peak VCO_2_(ml/min)	0.393	0.002*
AT (ml/min)	0.419	0.001*
VO_2_/WR slope (ml/min/watt)	0.339	0.008*
HR/VO_2_ slope (beats/ml/min)	0.363	0.004*
V_E_/VCO_2_ slope	−0.013	0.922

*CPET* Cardiopulmonary Exercise Test, *HRR* heart rate recovery, *FVC* forced vital capacity, *FEV*_*1*_ forced expiratory volume in the first second, *WR* work rate, *VO*_*2*_ oxygen uptake, *VCO*_*2*_ carbon dioxide production, *V*_*E*_ minute ventilation, *AT* anaerobic threshold

*significant difference, *P* < 0.05

**Table 4:** Multiple linearregression with HRR as the dependent variable

Coefficients						
Model		Unstandardized Coefficients		Standardized Coefficients	t	Sig.
		B	Std.Error	Beta		
1	(Constant)	12.641	3.783		3.342	.001
	using tiotropium	−4.327	2.005	−.265	−2.158	.035
	peak VCO_2_	.006	.002	.313	2.549	.014

## Discussion

The key finding of the present study was that HRR was significantly lower in COPD patients using tiotropium relative to those on no bronchodilator therapy. Further using tiotropium was an independent influencing factor for prediction of HRR in stable COPD patients. This result confirmed our hypothesis that anticholinergics would reduce vagal nervous activity.

HRR after CPET, which can be readily measured during exercise testing, is believed to be regulated, in part, by parasympathetic nervous system reactivation [11, 17], and as such a lower HRR therefore reflect slower parasympathetic activity. Compared with heart rate variability (HRV), HRR is easier to measure and interpret. Decreased parasympathetic input to the heart increases the potential for tachyarrhythmia and ischemia [5]^.^

COPD is linked to both sympathetic overactivation and autonomic dysfunction, with the latter being evident in the early stages of COPD [18]. Laccase et al. [12] demonstrated that COPD patients had lower HRR than normal controls, abnormal HRR was linked to poorer prognosis and increased all-cause mortality in COPD, although they did not observe a direct link between abnormal HRR and cardiovascular disease-related mortality owing to a lack of large number of cardiovascular deaths. Notably, cardiovascular disease, rather than respiratory failure, is the most common cause of death among those who have mild to moderate COPD [19, 20]. Our data showed that HRR was lower in the group taking tiotropium than in controls, suggesting that there maybe a higher cardiovascular risk in patients using tiotropium.

A study [21] reported that the heart rate deceleration capacity was reduced in patients with acute exacerbations of COPD (AECOPD) accompanied by ventricular tachycardia, reflecting an imbalance in cardiac autonomic regulation that potentially elevated the risk of sudden death. Rodríguez et al. [22] also found HRR following 6MWT independently predicted AECOPD over the follow-up period. Their study showed that patients with low HRR exhibited an elevated risk of AECOPD at 12 months following-6MWT assessment relative to those with higher HRR responses. Therefore, there is considerable evidence that in COPD patients, autonomic dysfunction is linked to poorer outcomes [12].

The first line treatment for COPD is generally anticholinergic agents, but these drugs are known to have the potential for increased cardiovascular risk. The Lung Health study [19], for example, revealed that inhaled ipratropium was linked with an elevated risk of supraventricular tachycardia owing to the fact that this drug is vagolytic. The mechanism may be that anticholinergic drugs cause autonomic disorders. Anticholinergics have two opposite effects on heart function [5]: suppression of parasympathetic control of heart rate and indirect reduction in sympathetic input to the heart. When the effect of anticholinergics on parasympathetic nerves is more intense, it increases the risk of tachyarrhythmia and ischemia. Gershon et al. [4] found that long-acting anticholinergics increased the risk of cardiovascular events, particularly within the initial 2–3 weeks of the therapy period. They hypothesized that this risk was related to the imbalance between direct and indirect cardiac actions during the initial 2–3 weeks period of drug use. Wu, et al. [23] enrolled 70 moderate-to-severe stable COPD patients, who were treated once per day with tiotropium for 3 months. They observed that the patients had significantly more low-frequency components and fewer high-frequency components of HRV (a correlate of autonomic imbalance) after tiotropium treatment for 1 month; however, they found no differences in HRV parameters at the 3-month assessment. These findings were consistent with the notion that early treatment lowers vagal modulation and increases sympathetic activity. We found that HRR was lower in patients who had used these agents for more than 1 year than in the control group. This may provide evidence that the longer-term usage of tiotropium affects autonomic balance, increasing the risk of adverse cardiac events.

Our analysis also showed that peak VCO_2_ and peak work rate were significantly lower in the patients using tiotropium≥1 year. These results may be related to the low mean FEV_1_ in the tiotropium using group.

The limitations of our study are that there was a lack of large number of COPD patients and we obtained only a short-term response. We could not stratify or compare different lung functional situations because of small sample size. Furthermore, the mechanism of the effect of tiotropium COPD was not investigated, but should be studied in the future. We intend to follow up the morbidity of cardiovascular (CV) events and cardiac death within 3 years. These CV events include heart failure, tachyarrhythmia, myocardial infarction and angina pectoris. This subsequent study will demonstrate whether these patients using tiotropium are at increased risk of adverse CV events.

## Conclusion

Stable COPD patients using tiotropium demonstrate reduced HRR, which may indicate alterations in cardiac autonomic function, and that maybe the reason for the aggravation of autonomic nervous imbalance. Therefore, we recommend that COPD patients taking anticholinergic bronchodilators should be considered for monitoring of cardiac function. Prescribers should be alert for potential cardiovascular events that may arise from autonomic nervous imbalance.

## Abbreviations

AECOPD: Acute exacerbations of COPD;
AT: Anaerobic threshold
BMI: Body mass index
BP: Blood pressure
COPD: Chronic obstructive pulmonary disease
CPET: Cardiopulmonary exercise test
CV: Cardiovascular
FEV_1_: Forced expiratory volume in the first second
FVC: Forced vital capacity
HR: Heart rate
HRR: Heart rate recovery
HRV: Heart rate variability
ICS: Inhaled corticosteroids
LABA/ICS: Combination of long-acting beta_2_-agonist plus corticosteroids in one device
LABAs: Long-acting beta_2_-agonists
LAMAs: Long-acting antimuscarinic antagonists
RCTs: Randomized clinical trials
SBP: Systolic blood pressure
SpO_2_: Oxygen saturation via pulse oximetry
VCO_2_: Carbon dioxide production
V_E_: Minute ventilation
VO_2_: Oxygen uptake
WR: Work rate
